# RETRACTION: Ultrapulse Carbon Dioxide Dot Matrix Laser for Facial Scar Treatment: A Meta‐Analysis

**DOI:** 10.1111/iwj.70321

**Published:** 2025-03-06

## Abstract

**RETRACTION:** YanH. , SunY. , HuY. , and WuY. , “Ultrapulse Carbon Dioxide Dot Matrix Laser for Facial Scar Treatment: A Meta‐Analysis,” International Wound Journal 21, no. 2 (2024): e14429, 10.1111/iwj.14429.PMC1082872437814494

The above article, published online on 09 October 2023, in Wiley Online Library (http://onlinelibrary.wiley.com/), has been retracted by agreement between the journal Editor in Chief, Professor Keith Harding; and John Wiley & Sons Ltd. Following an investigation by the publisher, all parties have concluded that this article was accepted solely on the basis of a compromised peer review process. The editors have therefore decided to retract the article. The authors did not respond to our notice regarding the retraction.

**RETRACTION:** H. Yan , Y. Sun , Y. Hu , and Y. Wu , “Ultrapulse Carbon Dioxide Dot Matrix Laser for Facial Scar Treatment: A Meta‐Analysis,” International Wound Journal 21, no. 2 (2024): e14429, 10.1111/iwj.14429.PMC1082872437814494

The above article, published online on 09 October 2023, in Wiley Online Library (http://onlinelibrary.wiley.com/), has been retracted by agreement between the journal Editor in Chief, Professor Keith Harding; and John Wiley & Sons Ltd. Following an investigation by the publisher, all parties have concluded that this article was accepted solely on the basis of a compromised peer review process. The editors have therefore decided to retract the article. The authors did not respond to our notice regarding the retraction.

